# Karyotype and genome size analyses for two spiders of the lycosidae family

**DOI:** 10.3389/fgene.2025.1544087

**Published:** 2025-03-25

**Authors:** Yuxuan Zhang, Mengying Zhang, Liang Leng, Ya Wu, Hanting Yang, Liangting Wang, Baimei Liu, Shuai Yang, Zizhong Yang, Shilin Chen, Chi Song

**Affiliations:** ^1^ School of Pharmacy, Chengdu university of Traditional Chinese Medicine, Chengdu, China; ^2^ Institute of Herbgenomics, Chengdu university of Traditional Chinese Medicine, Chengdu, China; ^3^ Yunnan Provincial Key Laboratory of Entomological Biopharmaceutical R&D, College of Pharmacy, Dali University, Dali, China

**Keywords:** Lycosidae, tissue drop technique, Hippasa lycosina, Lycosa grahami, karyotype, genome size

## Abstract

**Background:**

Karyotype and genome size are critical genetic characteristics with significant value for cytogenetics, taxonomy, phylogenetics, evolution, and molecular biology. The Lycosidae family, known for its diverse spiders with varying ecological habits and behavioral traits, has seen limited exploration of its karyotype and genome size.

**Methods:**

We utilized an improved tissue drop technique to prepare chromosome slides and compare the features of male and female karyotypes for two wolf spiders with different habits of Lycosidae. Furthermore, we predicted their genome sizes using flow cytometry (FCM) and K-mer analysis.

**Results:**

The karyotypes of female and male *Hippasa lycosina* were 2n♀ = 26 = 14 m + 12 sm and 2n♂ = 24 = 10 m + 14 sm, respectively, and were composed of metacentric (m) and submetacentric (sm) chromosomes. In contrast, the karyotypes of *Lycosa grahami* consisted of telocentric (t) and subtelocentric (st) chromosomes (2n♀ = 20 = 20th and 2n♂ = 18 = 12th + 6t, for females and males). The sex chromosomes were both X_1_X_2_O. The estimated sizes of the *H. lycosina* and *L. grahami* genomes were 1966.54–2099.89 Mb and 3692.81–4012.56 Mb, respectively. Flow cytometry yielded slightly smaller estimates for genome size compared to k-mer analysis. K-mer analysis revealed a genome heterozygosity of 0.42% for *H. lycosina* and 0.80% for *L. grahami*, along with duplication ratios of 21.39% and 54.91%, respectively.

**Conclusion:**

This study describes the first analysis of the genome sizes and karyotypes of two spiders from the Lycosidae that exhibit differential habits and provides essential data for future phylogenetic, cytogenetic, and genomic studies.

## 1 Introduction

The family Lycosidae is one of the largest within the order Araneae, with a global distribution, is known to comprise 2,474 species across 134 genera globally (https://wsc.nmbe.ch/accessed on 12 September 2024). Wolf spiders, as predatory species, hold significant research value in ecosystems, the economy, and scientific research. In the Arctic tundra ecosystem, they impact soil ecosystem functions through predation on other insects and small invertebrates, playing a crucial role in maintaining ecological balance (Koltz et al., 2018). The venom of Lycosidae spiders contains various bioactive substances with antitumor, antihypertensive, and antimicrobial properties, providing a rich resource for the development of new drugs (Ma et al., 2018; Reis et al., 2021; Tan et al., 2018). Furthermore, the chromosome number and karyotype characteristics of Lycosidae species exhibit significant diversity during evolution, which is of great importance for understanding genetic diversity and evolution (Araujo et al., 2015; Cavenagh et al., 2022).

**FIGURE 1:**
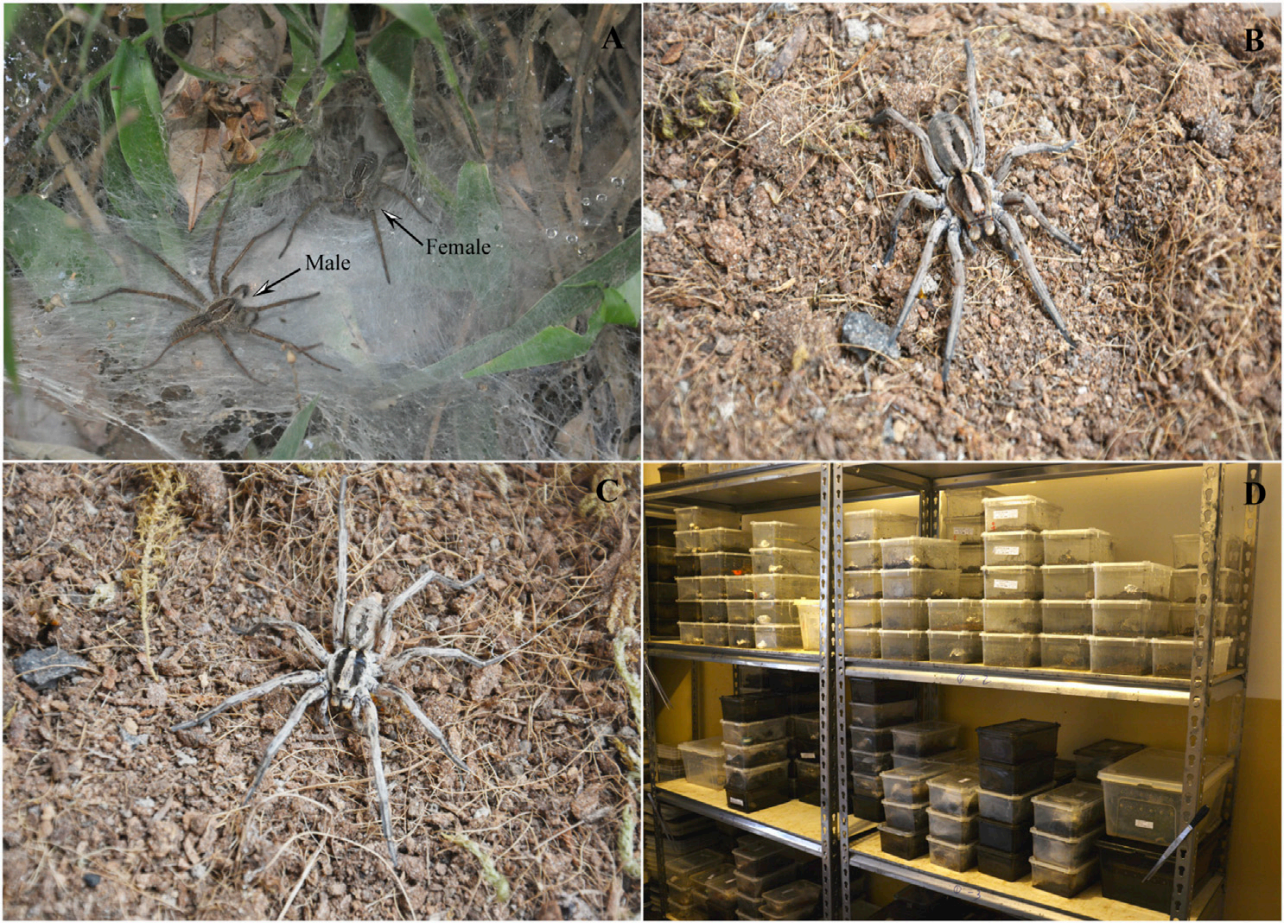
The habitats of the two spiders. **(A)** *H. lycosina* (large female and small male) are commonly found in rocky or soil crevices on slopes and spin large, irregular funnel webs with non-sticky, polygonal meshes. **(B)** An example of a female *L. grahami*. **(C)** An example of a male *L. grahami*; this wolf spider does not spin webs and frequents concealed areas such as rock crevices and soil cracks. **(D)** The feeding conditions provided in our laboratory. Note: The image was provided by Professor Zizhong from Dali University.

Numerous spiders have evolved web-building skills to facilitate prey capture, such as *Hippasa lycosina* (Figure 1A). In contrast, some spiders remain active hunters, relying on speed and fast-acting venom to subdue their prey, like *Lycosa grahami* (Figures 1B,C). These two distinct predatory strategies enable them to occupy different ecological niches, which may be related to species biodiversity (Koua et al., 2020). Differences in chromosome numbers and genomic characteristics are likely one of the important causes of biodiversity. In spiders, chromosome numbers range from 2n = 24 in *Trichonephila clavata* (♂) (Suzuki, 1954) to 2n = 94 in *Heptathela kimurai* (♂) (Datta and Chatterjee, 1988), and genome sizes range from 0.82 Gb in *Oedothorax gibbosus* (Hendrickx et al., 2022) to 6.79 Gb in *Macrothele yani* (You et al., 2024). These differences may be closely related to the evolutionary adaptability and ecological diversity of spiders (Ávila Herrera et al., 2021; Miles et al., 2024; Stávale et al., 2011).

The genome size and karyotype are pivotal cytogenetic characteristics that have been extensively utilized in taxonomic, phylogenetic, and evolutionary research (František et al., 2020; José Paulo da Costa Pinto et al., 2020; Král et al., 2019; Wayne et al., 2020). Chromosome karyotypes play a significant role in studying species systematics, relationships, origins, evolution, and classification (De Resende, 2017). However, previous karyotype studies in the Lycosidae have primarily focused on chromosome counts and sex chromosome behavior in males, with few comparative analyses between sexes (Araujo et al., 2015; Cavenagh et al., 2022; Chemisquy et al., 2008). To date, only 25 genera and 137 species within the family of Lycosidae have been investigated at the cytogenetic level (https://arthropodacytogenetics.bio.br/spiderdatabase/accessed on 12 September 2024) (Cavenagh et al., 2022; Chemisquy et al., 2008) (Supplementary Table S1). Diploid chromosome numbers in these species range from 18 to 30, with significant intrageneric variation. For example, there are 22–28 chromosomes in males of the *Hippasa* genera and 18 to 30 chromosomes in males from the *Lycosa* genera. Notably, the same species can exhibit multiple chromosome numbers, thus indicating potential aneuploidy, as reported for *H. deserticola* (2n♂ = 26/28) and *L. nigrotibialis* (2n♂ = 24/28) (Mittal, 1960; Mittal, 1963; Parida et al., 1986). Genome size (C-value) is a key measure of genetic information that is crucial for understanding genetic complexity and evolutionary history and is closely associated with ecological adaptation and evolutionary rates (Adachi et al., 2017; Olmo et al., 2002; Zuo et al., 2023).

Currently, the determination of genome sizes in plants and animals primarily relies on flow cytometry and genome survey sequencing. Flow cytometry stands as the gold-standard technique for genome-sizing in multicellular life forms (Wang et al., 2015). The accuracy of this technique depends on the availability of a species with a well-characterized genome size as an internal reference, ideally one with a genome size close to and stable in the target species (Dai et al., 2022). In the absence of ideal plant or animal standard references, existing data are largely based on subjectively selected standard species (Dolezel and Greilhuber, 2010). Despite the differences in cellular attributes between plants and animals, plants can effectively serve as internal references for animal genome size determination through meticulous experimental design and parameter optimization. For example, Hawlitschek et al. (2023) fine-tuned the nuclei lysis buffer composition and successfully determined the genome size of orthopteran insects using *Pisum sativum* (Fabaceae) as the internal standard. Similarly, Král et al. (2019) employed *Vicia faba* (Fabaceae) as the internal standard to estimate the genome sizes of various spiders. With the advancement of sequencing technology, genome survey sequencing can efficiently predict genome size, heterozygosity, and the proportion of repetitive sequences through k-mer analysis (Gao et al., 2022). Consequently, to improve genome-sizing accuracy, flow cytometry and genome survey sequencing are often combined for cross-validation before genome sequencing, enabling appropriate sequencing strategies (Gregory, 2005; Leng et al., 2024; Yang et al., 2024).

Previous research of the family of Lycosidae predominantly focused on taxonomy (Sankaran et al., 2017), biology (Naseem and Tahir, 2018), ecology (Persons and Rypstra, 2000; Xiao et al., 2015), and venom analysis (Abdel-Salam et al., 2021; Liu et al., 2021; Myshkin et al., 2019) and did not consider karyotype or genome size. Furthermore, previous research tended to focus more on male chromosomes, with no comparative analyses reported between sexes (Araujo et al., 2015; Cavenagh et al., 2022; Chemisquy et al., 2008; Kumbıçak et al., 2009). In the present study, we utilized flow cytometry and survey sequencing to determine the genome sizes of two spiders from the same family but with different predation strategies: *H. lycosina* and *L. grahami* and determine key indices, including genome size, heterozygosity, and repetitive sequences. In addition, we utilized the dropping technique for karyotype investigation. In undertaking this research, we aimed to establish a foundation for future cytogenetic and genomic studies in the family of Lycosidae.

## 2 Materials and methods

### 2.1 Specimen acquisitions

Adult male and female spiders were collected from the field and identified by Professor Zizhong (Table 1). The spiders were fed *Tenebrio molitor* regularly on a weekly basis and water was provided as needed to sustain their survival and maintain environmental humidity (Figure 1D). Voucher specimens were deposited at the Yunnan Provincial Key Laboratory of Entomological Biopharmaceutical R&D, Dali University, Dali. *Solanum lycopersicum* cv. Heinz 1706 was procured from Golden Future FCM Biotechnology Inc., and young leaves, approximately two months-of-age, were selected as an internal reference for flow cytometry. To eliminate potential discrepancies arising from variations in the number of sex chromosomes, female subjects were selected for the estimation of genome size (Král et al., 2019).

**TABLE 1:** Information relating to the collection of spider specimens.

Species	Number	Collection locality	Longitude	Latitude	Altitude (m)
*Hippasa lycosina* Pocock,1990	30♀, 50♂	Yangbi Yi Autonomous County, Yunnan Province, China	99°56′42″E	25°39′03″N	1718.1
*Lycosa grahami* Fox,1935	22♀, 20♂	Yuanma Town, Yuanmou X County, Yunnan Province, China	101°52′59″E	25°44′03″N	1140.0
*Trichonephila clavata* L. Koch, 1878	10♀	Wanqiao Town, Dali City, Yunnan Province, China	100°08′10″E	25°47′32″N	1937.7

### 2.2 Chromosome preparation

First, we performed a pre-experiment to optimize sampling, colchicine pretreatment, hypotonic time, and staining duration to generate metaphase plates from male *H. lycosina*, as described previously (Ávila Herrera et al., 2021). In brief, we acquired blood, legs, silk glands, gonads, and entire bodies of adult spiders. Samples were then pretreated with 0.01%–0.1% colchicine (prepared in RPMI 1640 complete medium) for 2 h at room temperature (RT). Samples were then centrifuged at 2000 rpm (5 min); then, the supernatants were removed, and the tissues were immersed in hypotonic solution (0.075 mol/L KCl) for 0.5–2 h at RT. Following re-centrifugation, the tissues were placed in freshly prepared and pre-cooled Carnoy’s solution (methanol: acetic acid (3:1) 4°C for 30 min; this procedure was repeated on two further occasions Subsequently, the tissues were transferred into 50% glacial acetic acid and dissociated for 5 min. To generate chromosome preparations, we employed the “dropping method” (Schmidt et al., 2023), a simple and efficient protocol, where a cell nuclear suspension is prepared and then dropped from a certain height onto a slide, causing the nuclei to burst and the chromosomes to spread. Finally, the slides were stained with 10% Giemsa (pH 6.8) for 10–30 min, rinsed three times with distilled water, dried naturally, and finally sealed with neutral resin. For the analysis of meiosis in males, the testes were dissected and preserved in Carnoy’s solution (methanol: acetic acid (3:1)) at 4°C. Chromosome smears were then prepared and stained with 10% Giemsa. Finally, observations and photographs were taken using an Olympus CX43 microscope (Olympus, Nagano, Japan) at ×100 magnification.

### 2.3 Karyotype analysis

To evaluate chromosomal morphology and construct karyotypes, 30 mitotic metaphases were acquired from the slides generated from each species. Five cells with clear morphology and well-dispersed mitotic metaphases were selected, and karyotype analysis software from Zeiss, Germany (Spot RT KE) was used to pair chromosomes and measure their lengths (short arm length and long arm length). Data were processed using Excel 2020 (Microsoft, Redmond, WA, United States) and mean values from 5 cells were used as parameters for karyotype analysis. Chromosome morphology was described and analyzed based on chromosome arm lengths and centromere positions, as described previously (Levan et al., 1964). The karyotype classification refers to Stebbins (Stebbins, 1971). The calculation formulas are as follows: Relative length (RL) = short arm length + short arm length), Arm ratio (AR) = (long arm length/short arm length), Chromosome length ratio (L/S) = longest chromosome length/shortest chromosome length, Karyotype asymmetry coefficient (As.K%) = (total length of long arms of chromosomes/total length of all chromosomes) × 100%.

### 2.4 Flow cytometry and the estimation of genome size

*S. lycopersicum* (2C = 0.85 pg (Sato et al., 2012)), a model species of the Solanaceae, was used as an internal reference. In order to ensure the reliability of our results, we used *Trichonephila clavata* (Araneidae) as a positive control; the genome for this species has already been reported (assembly size of 2.63 Gb and k-mer analysis of 2.72 Gb (Hu et al., 2023)). These experiments were performed with CyStain PI Absolute P (Seidl et al., 2022), spiders were sampled with legs, and *S. lycopersicum* was sampled with fresh leaves, all other procedures were identical. Samples were placed in 500 μL of nuclei extraction buffer and chopped with a sharp blade, after 60 s, the samples were filtered through a 50 μm filter, followed by incubation with 2000 μL of staining buffer containing RNase for 15 min in dark.

Stained samples were then analyzed with a CyFlow Cube6 flow cytometer (Sysmex Partec, Muenster, Germany) equipped with a 488 nm excitation light source. The fluorescence signals from at least 10,000 nuclei were acquired from each sample, and the coefficient of variation (CV) was controlled to within 5% to optimize reliability (Tomaszewska et al., 2021). Data were analyzed by FCSExpress (v5.0) software. Three biological replicate measurements were performed for each species to ensure the reliability of our results. Sample DNA content = (mean fluorescence intensity of sample/mean fluorescence intensity of the internal reference) × DNA content of the internal reference (Kerker et al., 1982). Genome size was estimated according to the conversion formula: 1 pg = 978 Mb (Dolezel and Bartos, 2005), and the mean of three measurements was taken to determine the final genome size.

### 2.5 K-mer analysis and the estimation of genome size

Genomic DNA was extracted from each sample using a modified Cetyltrimethylammonium bromide (CTAB)–based method (Aboul-Maaty and Oraby, 2019). Next, the DNA samples were randomly fragmented using a Covaris ultrasonic disruptor to construct sequencing libraries with fragment sizes of 150 bp. Subsequently, high-throughput sequencing was performed using DNBSEQ-T7 platform. Raw reads were filtered by FastQC (v0.20.1) software to remove low-density k-mers (<5) to minimize the impact of sequencing errors, thus resulting in clean reads, which were then used for subsequent analyses. To determine if there was any contamination in the sequencing data, we extracted the first 50,000 reads from the sequencing data and performed a blast alignment (v2.11.0+; parameters: evalue 1e-5 -max_target_seqs 1) with the Nucleotide Transcript Database (NT Database) (v202107). Species classification was conducted using MEGAN (v6.16.4).

Genome size was determined by GCE (v1.0.0.) This was performed by determining the k-mer frequency-depth distribution using Jellyfish (v2.2.10) and then estimating the genome size based on the k-mer frequency-depth distribution (Marçais and Kingsford, 2011). The genome size was calculated in accordance with a previous study as genome size = total number of k-mer/expected peak depth (Luo et al., 2023).

## 3 Results

### 3.1 Chromosome preparation and karyotype analysis

#### 3.1.1 Chromosome preparation

In this study, we used a range of biological specimens from *H. lycosina*, including blood, legs, silk glands, gonads, and entire bodies, to develop a chromosomal preparation method for the first time. Our analysis demonstrated that metaphase chromosomes were observed in the gonads with clear morphology but were either not observed in the other tissues or there was evidence of impurities, thus affecting observation (Supplementary Figure S1E). The optimal metaphase morphology was achieved when colchicine was used at a concentration of 0.05% and a treatment duration of 2 h (Supplementary Figure S2C). The best chromosome dispersion was achieved with 0.075 mol/L KCl solution for 1.5 h (Supplementary Figure S3C). In addition, we found that the best results were observed when samples were stained with 10% Giemsa solution for 20 min (Supplementary Figure S4B). Therefore, this optimized protocol was subsequently applied for chromosome preparation in other species.

#### 3.1.2 Karyotype analysis

**FIGURE 2:**
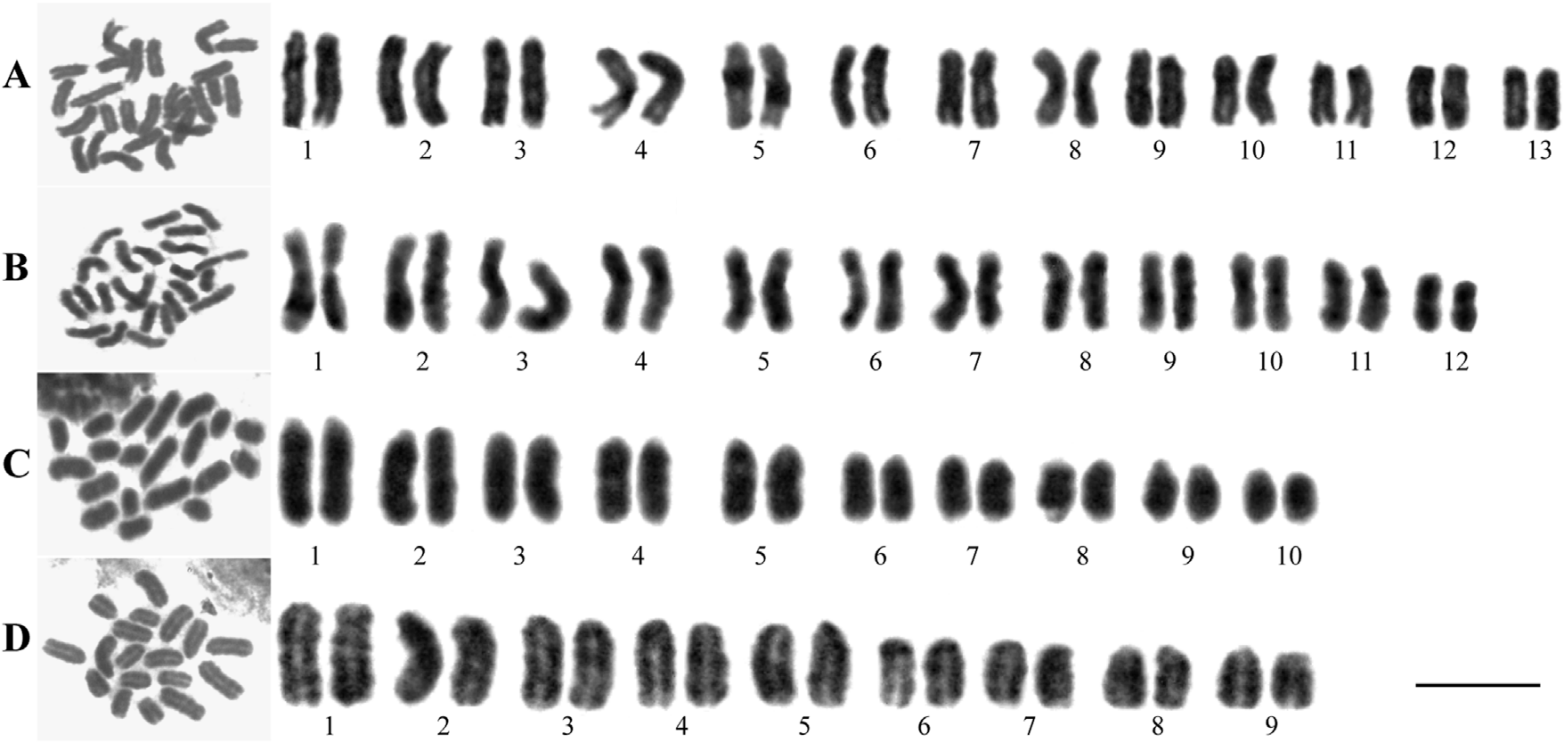
Metaphase mitotic chromosome (left) and karyotype arrangement diagrams (right) of two spiders. **(A)** Female *H. lycosina* (2n = 26). **(B)** Male *H. lycosina* (2n = 24). **(C)** Female *L. grahami* (2n = 20). **(D)** Male *L. grahami* (2n = 18). Bar = 20 μm.

This study is the first to reveal the chromosomal karyotypes of two wolf spiders from the Lycosidae family with different habits. The chromosome numbers were counted in female and male *H. lycosina* had 26 and 24 chromosomes, with 13 and 12 pairs of chromosomes. The female and male *L. grahami* were 20 and 18, paired with 10 and 9 pairs of chromosomes, respectively. Mitotic metaphase chromosomes are depicted in Figure 2 (Full pictures are provided in Supplementary Figure S5). The principal characteristics of two wolf spiders’ chromosomal karyotypes were presented in Table 2, with specific karyotypic parameters delineated in Supplementary Table S2. In two wolf spiders, the female spiders possess two additional chromosomes compared to the males, and both follow the sexual chromosome system (SCS) X_1_X_2_O. The *H. lycosina* chromosomes are predominantly metacentric (m) and submetacentric chromosomes (sm) (Figures 2A,B). Conversely, the *L. grahami* chromosomes are characterized mainly by the presence of subtelocentric (st) and telocentric chromosomes (t) (Figures 2C,D).

**TABLE 2:** Main karyotype characters of the two spiders.

Species	CL (μm)	ML (μm)	MAR	L/S	As. K%	KT	KF
*H. lycosina* (♀)	6.22–9.32	7.69	1.75	1.50	63.01	2A	14 m + 12 sm
*H. lycosina* (♂)	5.61–10.63	8.33	1.78	1.90	63.28	2A	10 m + 14 sm
*L. grahami* (♀)	6.91–13.74	10.00	3.22	1.99	96.30	4A	20st
*L. grahami* (♂)	8.30–14.19	11.11	6.93	1.71	86.15	4A	12st+6t

CL, chromosome length range; ML, mean chromosome length; MAR, mean arm ratio; L/S: the ratio of the longest to the shortest chromosome length; As.K%, karyotype asymmetry coefficients; KT, karyotype type; KF, karyotype formula.

**FIGURE 3:**
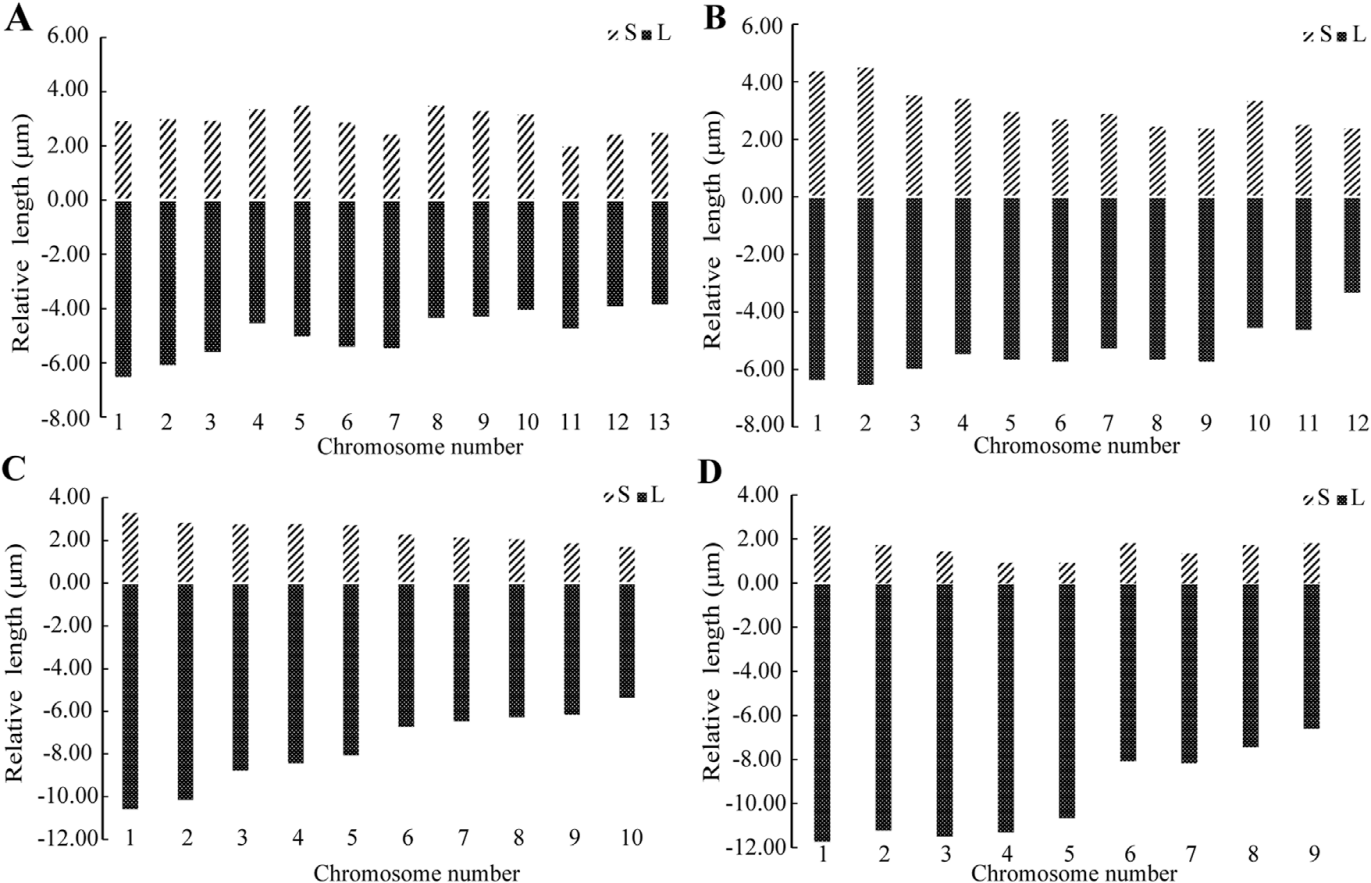
Karyotype diagrams for the two spiders. **(A)** Female *H. lycosina* (2n = 26, 14 m + 12 sm). **(B)** Male *H. lycosina* (2n = 24, 10 m + 14 sm). **(C)** Female *L. grahami* (2n = 20, 20th). **(D)** Male *L. grahami* (2n = 18, 12th + 6t). Positive and negative values indicate short and long arms, respectively.

A comparison of the chromosome lengths between male and female *H. lycosina* revealed that the mean length (7.69 μm) and length range (6.22–9.32 μm) of female chromosomes were shorter than those of males, which had a mean chromosome length of 8.33 μm and a length range of 5.61–10.63 μm). Comparison of the arm ratio (the length of the long arm/short arm, AR) of female and male chromosomes revealed that the mean AR (1.78) and AR range (1.23–2.44) in males were greater than the mean AR (1.75) and AR range (1.25–2.42) of females (Supplementary Table S2)). To compare the length of each chromosome more intuitively, the long arm and short arm of the chromosomes from male and female spiders were presented as bar charts (Figures 3A,B). The largest chromosomes in females and males were submetacentric (sm) and metacentric chromosomes (m), with lengths of 9.32 μm and 10.63 μm, respectively. The shortest chromosomes were all metacentric chromosomes (m), with lengths of 6.22 μm in females and 5.61 μm in males. In *H. lycosina*, karyotype asymmetry coefficients (As.K%) were 63.01% in females and 63.28% in males. The length ratios of the longest to shortest chromosomes (L/S) were 1.50 in females and 1.90 in males. The proportion of chromosomes with an arm ratio greater than 2:1 was 0.15 in females and 0.13 in males. The karyotype classification for both female and male *H. lycosina* was type 2A.

In a comparative study of the chromosome lengths in female and male *L. grahami*, we observed that the mean length of chromosomes in females (10.00 μm) was less than that in males (11.11 μm), although the chromosome length range in females (6.91–13.74 μm) was broader than that in males (8.30–14.19 μm). Comparison of the AR of chromosomes in females and males revealed that the mean AR (6.93) and range of AR (3.74–12.20), in males exceeded those in females, which were 3.22 and 3.02–3.65, respectively (Supplementary Table S2). For a more direct comparison of the length of each chromosome, we generated bar graphs (Figures 3C,D) to represent the long and short arms of the chromosomes in male and female *L. grahami*. The largest and smallest chromosomes of female and male spiders were subtelocentric chromosomes (st). The lengths of the large chromosomes were 13.74 μm and 14.19 μm, respectively, while the lengths of the small chromosomes were 6.91 μm and 8.30 μm, respectively. In *L. grahami*, females and males exhibited As.K% values of 96.30% and 86.15%, respectively, with L/S of 1.99 and 1.71. All chromosomes had arm ratios exceeding 2:1. The karyotypes of both females and males were classified as type 4A.

**FIGURE 4:**
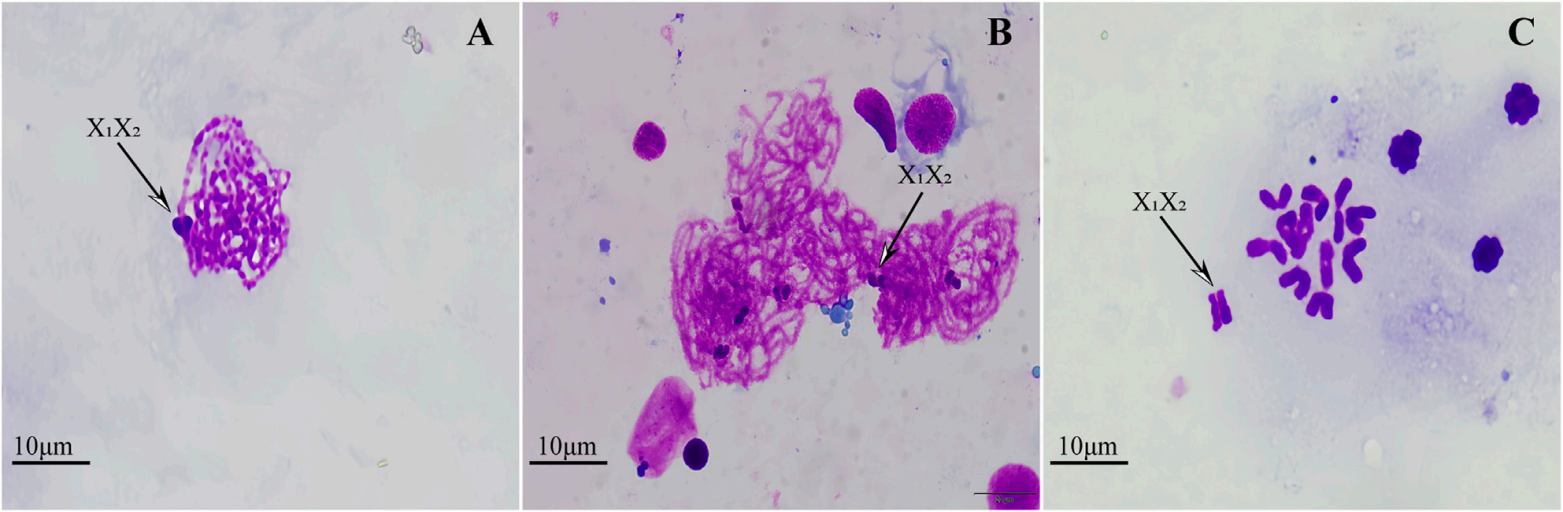
Meiotic cells in the two spiders, male. **(A, C)** *H. lycosina*. **(B)** *L. grahami*. **(A, C)** Cells from the two spiders in the pachytene stage of meiosis exhibited positive heteropycnotic sex chromosomes. **(C)** Diakinesis cells confirmed the number of bivalents and sex chromosomes in *H. lycosina* with 11 autosomal bivalents and X_1_X_2_. The arrow represents the sex chromosome. Bar = 10 μm.

To demonstrate the existence of sex chromosomes in the two spiders, we conducted meiotic observations on males from each species. The analysis showed that in the meiotic cells of both male species, the sex chromosomes could be readily discerned during the pachytene nucleus due to their extreme condensation condensation and positive heteropycnosis (Figures 4A,B). Diakinesis cells confirmed the number of bivalents and sex chromosomes in *H. lycosina* with 11 autosomal bivalents and two sexual univalents (n = 11 + X_1_X_2_) (Figure 4C).

### 3.2 Genome size analysis

#### 3.2.1 Genome size estimation by flow cytometry

**FIGURE 5:**
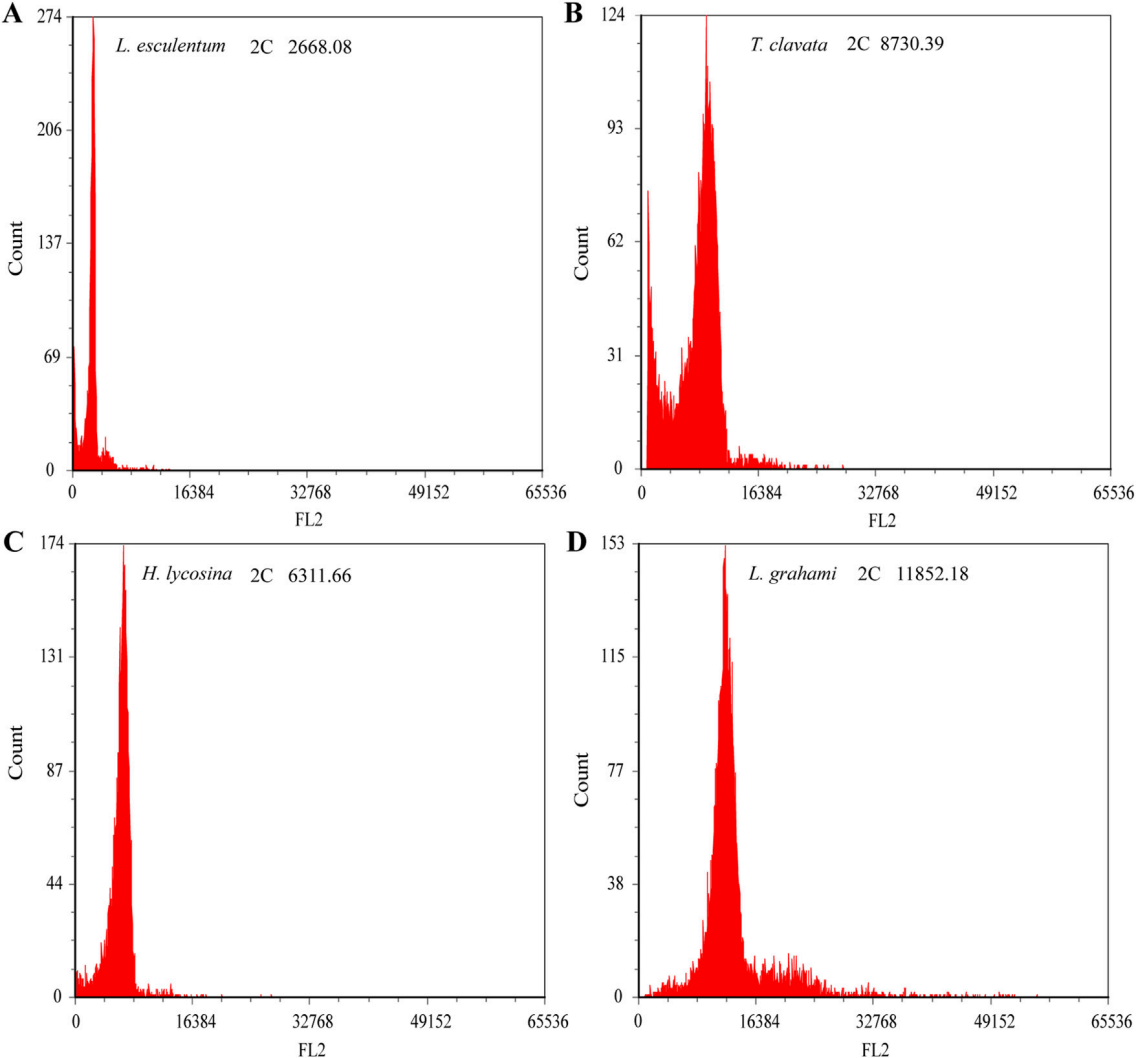
Histogram shows 2C DNA content in the two spiders, as determined by flow cytometry. The X-axis represents the relative fluorescence intensity of nuclei stained with PI in the nuclear suspension, while the Y-axis indicates the number of nuclei. **(A)** *S. lycopersicum* was used as the internal reference (2C = 0.85 pg, 2C peak channel is 2668.08). **(B)** *T. clavata* (2720.15 Mb, 2C peak channel is 8730.39). **(C)** *H. lycosina* (1966.54 Mb, 2C peak channel is 6311.66). **(D)** *L. grahami* (3692.81 Mb, 2C peak channel is 11852.18).

The genome sizes for the two spiders were estimated by FCM. *S. lycopersicum* was used as an internal reference and *T. clavata* was used as positive control. Three biological replicates of measurements were performed for each species and results exhibited good reproducibility. Analysis predicted that the size of the *T. clavata* genome was 2720.15 Mb, which was consistent with the genome assembly. The mean fluorescence intensities were 6311.66 and 11,852.18 for *H. lycosina* and *L. grahami*, respectively (Figure 5). The calculated genome sizes were 1966.54 Mb and 3692.81 Mb for *H. lycosina* and *L. grahami*, respectively (Table 3).

**TABLE 3:** Genome sizes for the two spiders, as estimated by flow cytometry.

Species	Fluorescence intensity of internal reference	Fluorescence intensity of sample	Fluorescence intensity ratio	C-value/pg DNA-2C	Genome size (Mb)
*H. lycosina*	2668.08	6311.66	2.37	2.01	1966.54
*L. grahami*	2668.08	11,852.18	4.44	3.78	3692.81

#### 3.2.2 Genome size estimation by K-mer analysis

Next, we used the DNBSEQ-T7 high-throughput sequencing platform to analyze *H. lycosina* and *L. grahami* using small insert size library sequencing. After filtering low-quality sequencing reads, the total number of bases in the *H. lycosina* and *L. grahami* genomes was determined to be 454.88 Gb (Q30 95.40%) and 335.26 Gb (Q30 93.37%), with a GC content of approximately 32.45% and 32.17%, respectively (Supplementary Table S3). Analysis of the distribution of GC content revealed that there was no separation between AT and GC in the sequenced sequence (Supplementary Figure S6). Next, 50,000 reads were extracted from the filtered data and compared to the NT database (Supplementary Table S4). Comparative analysis revealed that there was no significant DNA contamination from external sources. Collectively, our findings suggested that the sequencing quality was excellent and could be used for survey analysis as well as subsequent whole-genome sequencing.

**FIGURE 6:**
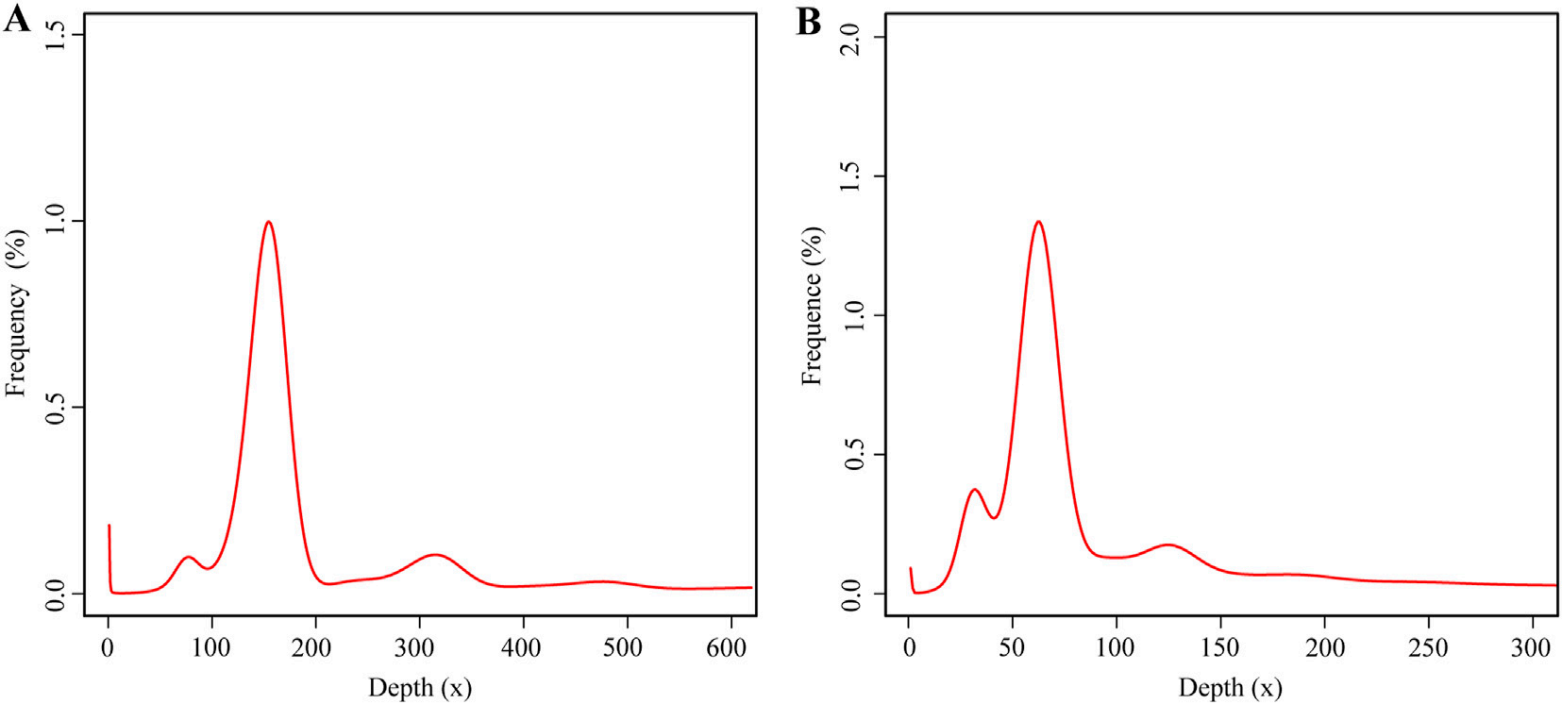
19-mer frequency distribution graph of sequencing reads for the two spiders. The X-axis represents the sequencing depth, while the Y-axis represents the ratio of specific k-mer to the total number of k-mer for a given sequencing depth. **(A)** Distribution curve of *H. lycosina*. **(B)** Distribution curve of *L. grahami*.

Based on the 19-mer frequency distribution, the genome size of *H. lycosina* was estimated to be 2099.89 Mb (Table 4), 1.07-fold larger than that of the size estimated by FCM (1966.54 Mb); the heterozygosity and duplication rates were 0.42% and 21.39%, respectively (Figure 6A). The estimated size of the *L. grahami* genome was 4012.56 Mb (Table 4), 1.09-fold larger than the size estimated by FCM (3692.81 Mb), with heterozygosity and duplication rates of 0.80% and 54.91% (Figure 6B). Both the rates of heterozygosity and duplication in the *L. grahami* genome were larger than those in the *H. lycosina* genome.

**TABLE 4:** 19-mer analysis for the estimation of genome size in the two spiders.

Sample	K-mer number	K-mer depth	Genome size (Mb)	Heterozygous ratio (%)	Duplication ratio (%)
*H. lycosina*	340,518,852,100	161.79	2099.89	0.42	21.39
*L. grahami*	247,773,629,878	61.68	4012.56	0.80	54.91

## 4 Discussion

### 4.1 Chromosome preparation

High-quality mitosis metaphase cells are essential for chromosome karyotype analysis. In the field of spider chromosome research, seven methods for preparing chromosome slides of spiders have been documented and experimentally verified: reproductive gland section, testis squash, single embryo drop, mixed embryo drop, blood cell preparation, single embryo squash, and needle smear technique. However, these methods have many drawbacks in practical application. The reproductive gland section technique has a low detection rate, and there are variations in chromosome morphology and number, which is now rarely used. The testis squash technique is currently mainly used for preparing meiotic chromosome spreads (Král et al., 2022). The single embryo drop technique generally yields unsatisfactory results (Matsumoto, 1977). The mixed embryo drop technique yields better results, but it is prone to chromosome loss during staining, and the significant differences in oviposition times among different spiders make it difficult to collect materials (Hu et al., 2023). The blood cell chromosome preparation method yields poor results, as there are large individual variations in spider size, and spider blood is scarce, making it difficult to collect materials. The needle smear method involves feeding spiders with colchicine and then preparing slides from the gonads, which yields relatively ideal results, but the experimental cycle is relatively long, making it difficult to control variables. Therefore, in this study, we optimized chromosome preparation methods for two species of spiders, including sampling, pretreatment, and hypotonic treatment steps, and successfully generated metaphase chromosomes for karyotype analysis. Compared to traditional methods, the approaches used in this study are more straightforward to operate and offer better reproducibility. Tissues with high mitotic activity are preferred for karyotype preparations as these ensure a sufficient number of mitotic phases (Indy et al., 2010).

In Araneae, various tissues have been found to be suitable for chromosome preparation, including embryos, blood, abdominal tissues, and gonads (Wayne et al., 2020). In the present study, we identified gonads as the optimal tissue for metaphase spread acquisition, as other tissues yielded fewer mitotic phases, potentially due to a lower mitotic index. Furthermore, pretreatment is a crucial step when generating chromosome preparations; consequently, the dose of colchicine and the processing time are important factors that can affect chromosome preparation. The role of this treatment is to impede the formation of the spindle, prompting the chromosomes to shorten and thicken, thus generating metaphase spreads with suitable chromosome morphology (Tianwen et al., 2020). Furthermore, the complete dissection of gonads is known to have a significant impact on chromosome preparation.

### 4.2 Karyotype analysis

In this research, we conducted comparative karyotype analysis of *H. lycosina* and *L. grahami*, two species of spider from Yunnan, China. The analysis revealed that the chromosome number of diploids and SCS observed in the two spiders were consistent with those previously reported for the congeneric species *H. agelenoides* and *Lycosa* sp.: 2n♂ = 24 and 2n♂ = 18, X_1_X_2_O-X_1_X_2_ (Sharma et al., 1958; Srivastava and Shukla, 1986). The Lycosidae family exhibits considerable diversity in chromosome number, with male diploid numbers (2n) ranging from 18 (in *L. narborensis*, Lycosa sp., and this study) to 28 (in *Alopecosa aculeata*, *L. bistriata*, and *Arctosa cinerea*, among others) (Supplementary Table S1). Notably, a chromosome number of 28 has been detected in over 50% of species; this potentially represents the modal diploid number within the family (Chemisquy et al., 2008). However, many Lycosidae species still exhibit lower diploid numbers (2n♂ < 28) (Supplementary Table S1, S5, and this study). It is worth noting that a chromosome number of 18 is the lowest ever reported in the family Lycosidae and has only been only reported in the genera *Lycosa*. Previous studies have hypothesized that the ancestral male karyotype of wolf spiders consists of 28 acrocentric chromosomes (Bugayong et al., 1999; Datta and Chatterjee, 1989; Dolejš et al., 2011; Sharma et al., 1958). In some Lycosidae lineages, a gradual reduction in diploid chromosome number has occurred, likely due to centromere fusion, a phenomenon that is also observed in other entelegyne spiders (Suzuki, 1954). Except for the ancestral number of 2n = 28, chromosome number reductions are particularly evident in the Lycosinae, Evippinae, and Venoniinae subfamilies (Dolejš et al., 2011).

We identified significant differences in chromosome number and morphology between the two spiders, indicating that these two species possess distinct genetic characteristics. In addition, a chromosomal discrepancy existed between the sexes of these spiders. We found that males had two fewer chromosomes than females. This disparity, along with variations in karyotypic formulae, morphology, and size, may correlate with SCS differences. In the Lycosidae family, the SCS has been classified as XO (Araujo et al., 2015), X_1_X_2_O, and X_1_X_2_X_3_O (Brum-Zorrilla and Postiglioni, 1980). Our study confirmed that both species were X_1_X_2_O SCS, which is prevalent among the Lycosidae. Typically, 95% of Lycosidae species have an SCS of X_1_X_2_O-X_1_X_2_/X_1_X_1_X_2_X_2_ (male/female), in which O indicates the absence of a Y chromosome, and the male sex chromosome consists of two X chromosomes and the female sex chromosome consists of two pairs of X chromosomes (Chemisquy et al., 2008). This SCS is the ancestral type of spiders including the Lycosidae (Datta and Chatterjee, 1989; Dolejš et al., 2011; Suzuki, 1954). However, sex chromosomes varied in length among the species of Lycosidae studied thus far, including the largest, medium-sized, and smallest length of chromosomes (Araujo et al., 2015; Cavenagh et al., 2022; Dolejš et al., 2011). These differences may be due to sex chromosome and/or autosomal rearrangements that result in variations in their size. Regrettably, our study could only confirm the presence of two sex chromosomes in male specimens during specific meiotic phases, making it impossible to determine the exact location and size of the sex chromosomes.

### 4.3 Genome size analysis

The genome of only species from the Lycosidae, *Pardosa pseudoannulata*, has been reported previously; this genome was 2420 Mb in size and had high levels of heterozygosity (2.77%) (Yu et al., 2024). The size of the *H. lycosina* genome generated in this study was 1966.54–2099.89 Mb and had low levels of heterozygosity and repetition. In addition, the size of the *L. grahami* genome was 3692.81–4012.56 Mb and had higher levels of heterozygosity and repetition. In addition, we also found that the genome size predicted by flow cytometry was slightly lower than that predicted by the genome k-mer analysis. This may have been caused by the flow cytometry technique being affected by factors such as sampling site, internal reference selection, and the processing environment when determining genome size (Dai et al., 2022). Of the two methods used to determine genome size in this study, flow cytometry is by far the most commonly used method, predominantly because it is low cost and fast, but this method also requires expensive instruments and internal references (Del Mar Ochoa-Saloma et al., 2020; He et al., 2016; Liu et al., 2016; Pflug et al., 2020; Swathi et al., 2018). The selecting of the plant *S. lycopersicum* as an internal reference in this study is based on its stable and well-defined genome size, which has been widely used in flow cytometry studies (Cerca et al., 2022). To further verify the reliability of *S. lycopersicum* as an internal reference, this study also employed *T. clavata*, a species with a well-characterized genome size, as a positive control (Hu et al., 2023). The result indicated that the flow cytometry-predicted genome size of *T. clavata* (2720.15 Mb) was essentially consistent with the published value. It suggests that *S. lycopersicum* can serve as an effective internal reference species for predicting the genome sizes of the two wolf spiders. K-mer analysis can yield rich and accurate information and can predict genome size, heterozygosity, the repetitive sequence ratio, and GC content. However, it can be easily affected by multiple factors, such as data quality, the selected software, and the parameter settings (Baeza et al., 2021; Fan et al., 2022). Therefore, these methods are often used in combination to provide a comprehensive judgment when estimating the genome size of a species within a relatively accurate range (Luo et al., 2023; Mgwatyu et al., 2020; Zhou et al., 2023).

Generally, higher-level organisms possess more complex genetic information than lower-level organisms, which might suggest that higher-level organisms have larger genome sizes. However, there is no strict correlation between genome size and the complexity of organisms (the C-value paradox) (Kim et al., 2014). This is because genomes contain many highly repetitive DNA sequences, resulting in a conflict between DNA content and evolutionary level. In a previous study of the Araneae, the genome sizes of 26 spiders were determined by FCM, ranging from 1.7 to 4.7 Gb (Král et al., 2019). In contrast, the genome sizes of spiders predicted by k-mer analysis ranged from 0.7 Gb in *O*. *gibbosus* to 6.50 Gb in *Acanthoscurria geniculata* (Sanggaard et al., 2014; Wang et al., 2022). These previous studies highlight the substantial variation in DNA content even among closely related species. Spiders, as an ancient and highly diverse group of animals, exhibit considerable variation in genome size and karyotype. These differences may be closely related to the different ecological habits, living environments, and evolutionary histories of the species (Lertzman-Lepofsky et al., 2019; Liedtke et al., 2018; Saha et al., 2023).

To investigate the potential link between the genome sizes and karyotypes of spiders, we collected existing data relating to the genome size and karyotype of 16 spider species and performed a comparative analysis. The results revealed significant differences in genome size and chromosome number both among different families and within the same family. Moreover, no significant correlation was found between genome size and chromosome number (Supplementary Figure S7). A previous study detected chromosome numbers in 6,052 genome size records and found no significant correlation between genome size and chromosome number (El Shehawi and Elseehy, 2017). However, due to the limited sample size in our study, which only investigated the karyotypes and genome sizes of two spider species, the generalizability of our findings is constrained. Consequently, further research and validation are essential to better understand the diversity and evolutionary history of Lycosidae species.

This study was the first preliminary study exploring the karyotype and genome size of two wolf spiders with distinct habits, both belonging to the Lycosidae. We found that these spiders exhibited significant differences in genome sizes, chromosome numbers, morphologies, and sizes, potentially reflecting their unique genetic traits and evolutionary histories. Karyotype analysis revealed that the webbing *H. lycosina* primarily has metacentric and submetacentric chromosomes, while the hunting *L. grahami* features predominantly acrocentric and telocentric chromosomes; the SCS was X_1_X_2_O. The genome of *H. lycosina* was relatively small, with low levels of heterozygosity and repetition, while the *L. grahami* genome was larger with higher levels of heterozygosity and repetition. Collectively, our findings provide novel insights into the karyotype, genome size, heterozygosity, and repetitive sequence proportions of the two spiders, yielding valuable data for further research in cytogenetics, taxonomy, phylogeny, and whole-genome sequencing of Lycosidae.

## Data Availability

The data used in this study can be found in the article/Supplementary Materials.
