# Supplementary material for: Karyotype and genome size analyses for two spiders of the lycosidae family
Source: Front Genet. 2025 Mar 25;16:1544087. doi: 10.3389/fgene.2025.1544087 (PMC11975668; doi:10.3389/fgene.2025.1544087)
Supplement: Supplementary file 1 [file DataSheet1.pdf]

Supplementary Material

Supporting Figures

Figure legends

Figure S1 Chromosome morphology of different tissues in male *H. lycosina*.

Figure S2 Chromosome morphology of testes from *H. lycosina* following treatment with different concentrations of colchicine.

Figure S3 Chromosome morphology of testes from *H. lycosina* following different durations of hypotonic treatment different hypotonic times.

Figure S4 Chromosome morphology of testes from *H. lycosina* following different staining durations.

Figure S5 Metaphase chromosomes in the two spiders.

Figure S6 GC content distribution in the two species of spiders.

Figure S7 Relationship between genome size and chromosome number in sixteen spiders.

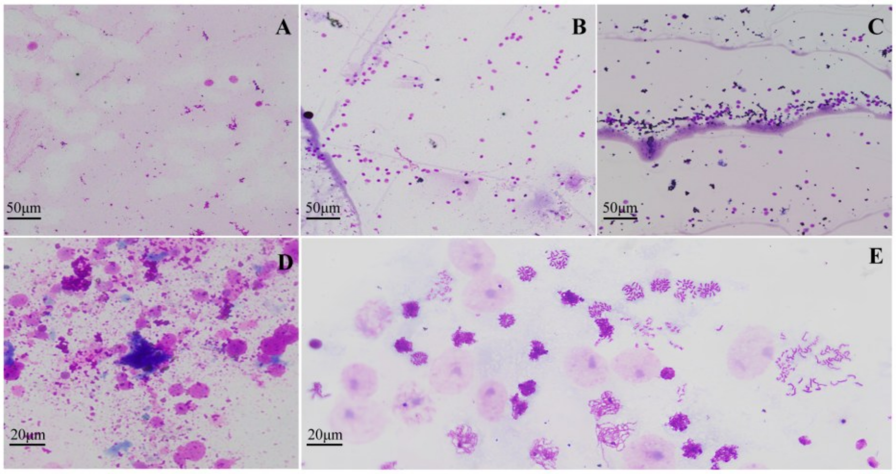

Figure S1 Chromosome morphology in different organs from male *H. lycosina*.
(A) Blood. **(B)** Legs. **(C)** Silk glands. **(A-C)** Few cells showing the absence of mitotic metaphase chromosomes. **(D)** Entire organism, abundant cells but few mitotic metaphase chromosomes, with excessive impurities resulting in blurry and indistinguishable chromosomes. **(E)** Gonads (the male testis and female ovary), exhibiting numerous mitotic metaphase chromosomes, featuring well-formed chromosomes that were easily observable. Bar = 20 μm and 50 μm.

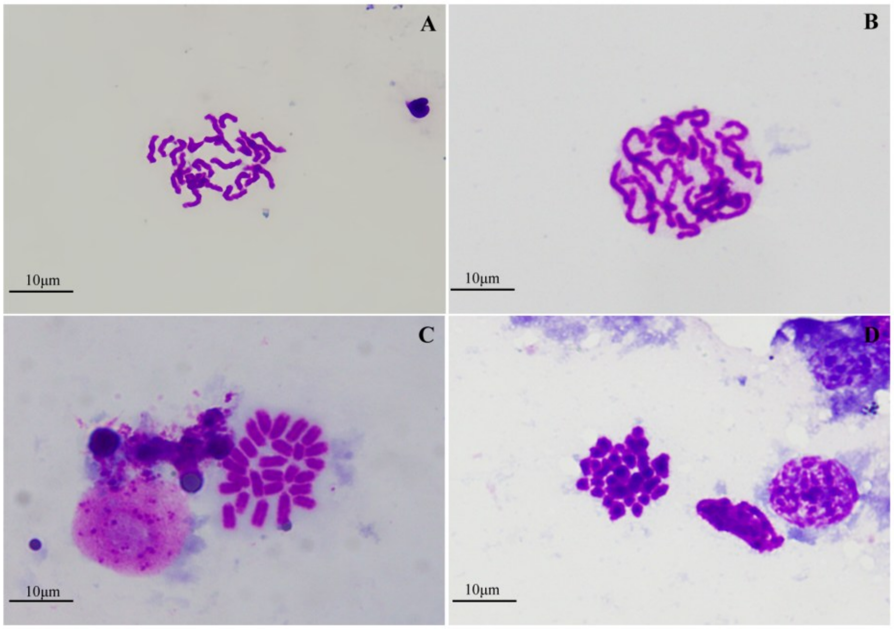

Figure S2 Chromosome morphology of testes from *H. lycosina* following treatment with difference concentrations of colchicine.
(A-D) Pretreatment with colchicine (0.01%, 0.03%, 0.05%, and 0.1%) for 2 hours. **(A)** Chromosomes appeared elongated and adhered, making identification difficult. **(B)** Chromosomes adhered and overlapped, resulting in poor resolution. **(C)** Chromosomes were shorter, thicker, and exhibited clear morphology, with good dispersal, facilitating easy identification. **(D)** Chromosomes aggregated into punctate forms, displayed poor dispersion, and were challenging to observe. Bar = 10 μm.

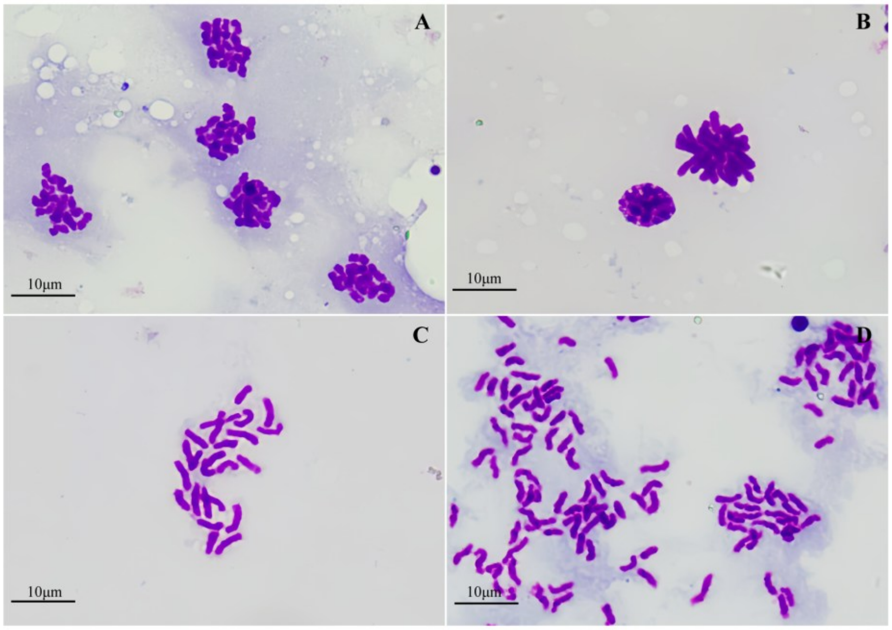

Figure S3 Chromosome morphology of testes from *H. lycosina* following different durations of hypotonic treatment.
(A-D) Hypotonic treatment with 0.075mol/L KCl solution for 0.5, 1, 1.5 and 2 hours, respectively. **(A, B)** Chromosomes were stacked together, poorly dispersed and difficult to recognize. **(C)** Chromosomes were well dispersed and easily recognized. **(D)** Chromosomes were over-dispersed and not easily counted. Bar = 10 μm.

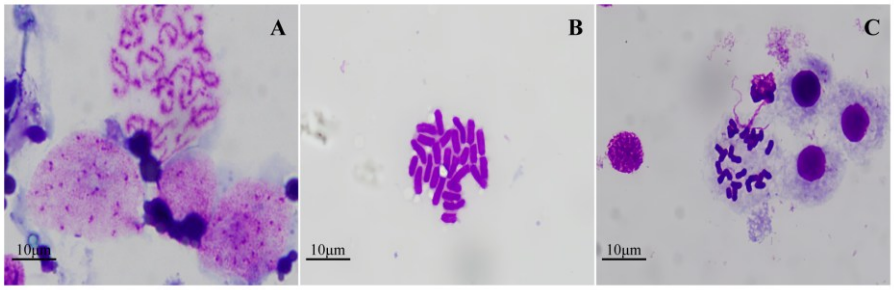

Figure S4 Chromosome morphology of testes from *H. lycosina* after different durations of staining
(A-C) 10% Giemsa staining of the gonads for 10, 20, and 30 min, respectively.
(A) The chromosomes were lightly stained, making it difficult to observe the number and morphology of chromosomes. **(B)** The chromosomes were well-stained, achieving the best effect, and it was easy to observe the number and morphology of chromosomes. **(C)** The chromosomes were deeply stained, making it difficult to distinguish their morphology and observe them clearly. Bar = 10 μm.

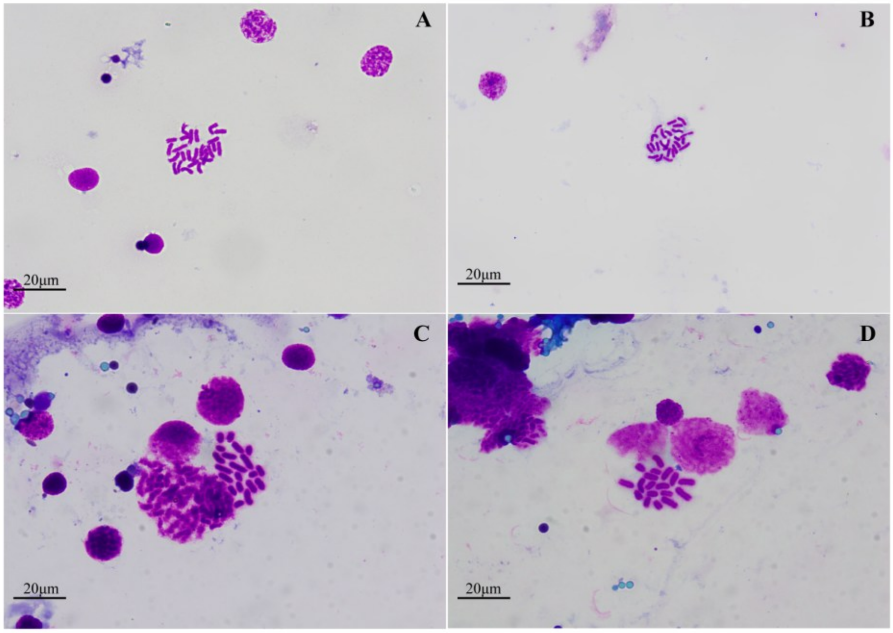

Figure S5 Metaphase chromosomes in the two spiders.
(A) Female *H. lycosina* (2n = 26). **(B)** Male *H. lycosina* (2n = 24). **(C)** Female *L. grahami* (2n = 20). **(D)** Male *L. grahami* (2n = 18). Bar = 20 μm.

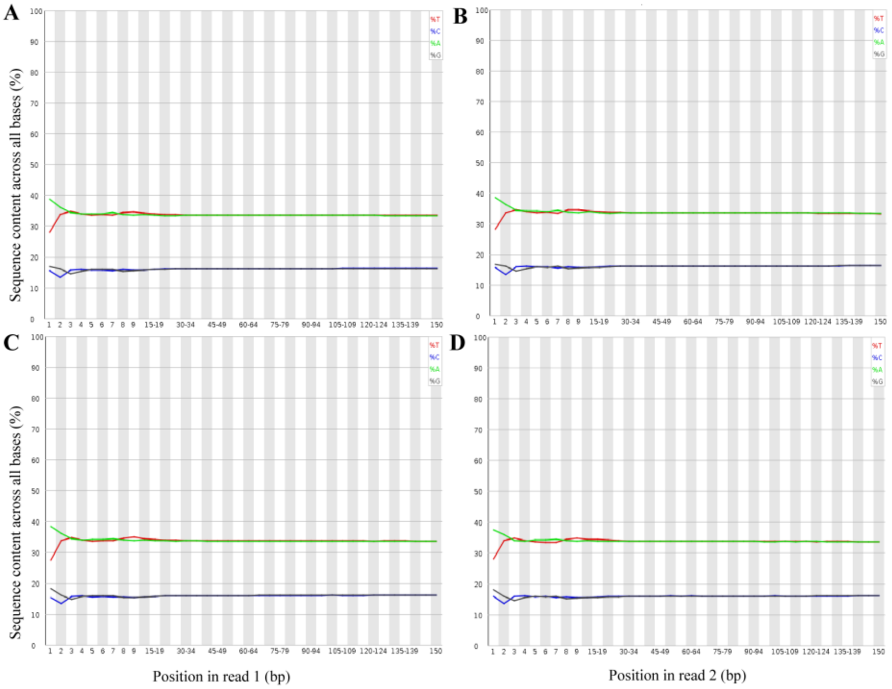

Figure S6 GC content distribution in the two spiders.
The distribution of GC content in sequencing reads (read-1 and read-2) of *H. lycosina* **(A, B)** and *L. grahami* **(C, D).** The proportions of A, G, C, and T were similar, with no significant GC bias, thus indicating good sequencing quality.

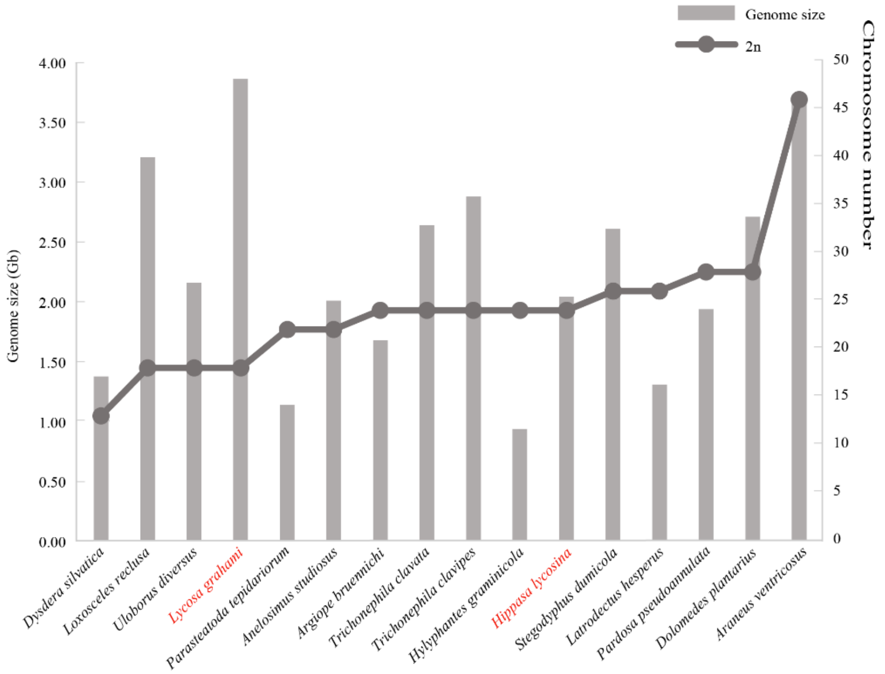

Figure S7 Relationship between genome size and chromosome number in sixteen spiders. The bar graph shows the genome size for each species, with the height of the bar corresponding to the value of the genome size. The line graph, on the other hand, shows the number of chromosomes (2n) for each species, with the points on the line corresponding to the numerical value of chromosome number. Red text indicates the two species from this study. Genome sizes of the two species in this study were expressed as the average of flow cytometry and K-mer analysis results. Analyzed data from Table S6 and S7.
